# Spectrum of Congenital Heart Diseases in Iraqi Patients

**DOI:** 10.5152/eurasianjmed.2026.251236

**Published:** 2026-02-23

**Authors:** Sadiq M Al-Hamash, Amer Naes Amer, Kamal Ali Mohammed Maerozy, Khalid A. Khalid

**Affiliations:** 1 Ibn-Albitar Center for Cardiac Surgery, Baghdad, Iraq; 2 University of Duhok College of Medicine, Duhok, Iraq; 3 Alsayyab Cardiac Center, Basra, Iraq

**Keywords:** Congenital anomalies, congenital heart defects, prevalence

## Abstract

**Background:**

Early detection and risk stratification of congenital heart disease (CHD) are of utmost importance for timely management. This study aimed to report on CHD among patients in Baghdad, Iraq.

**Methods:**

This prospective study was conducted from September 1, 2022 to September 1, 2023 among 2221 patients of all age groups diagnosed with CHD.

**Results:**

This study analyzed 2221 patients with CHD, of whom 76.7% had acyanotic CHD and 23.3% had cyanotic CHD. The mean age was higher for acyanotic cases (81.3 months) than for cyanotic cases (25.6 months). Almost half (47.5%) of the diagnoses were made in infancy, and 10.9% were made in the neonatal period. Only 13.2% of the diagnoses were made in adulthood. Ventricular septal defect (VSD) was the most frequent lesion, mainly diagnosed in infancy, whereas atrial septal defect (ASD) was often recognized in adulthood (42%). Critical conditions such as D-transposition of great arteries and hypoplastic left heart syndrome were mostly neonatal diagnoses. Gender distribution was balanced overall, though ASD and patent ductus arteriosus were significantly more common in females, while aortic stenosis, D-transposition of the great arteries, and coarctation predominated in males. Ventricular septal defect (30.2%) was the leading acyanotic lesion, while Tetralogy of Fallot (9.4%) was the most common cyanotic CHD.

**Conclusions:**

This research indicates that acyanotic CHD prevails in Iraq, with VSD being the most common. More than half of the sample was diagnosed by the age of 2. A male predominance was observed throughout, indicating a need for early screening with gender consideration.

**Main Points**

- Acyanotic vs. cyanotic prevalence and age: Acyanotic congenital heart disease (CHD) was quite a bit more prevalent than cyanotic CHD and was identified at a significantly greater mean age.
- Early diagnosis is dominant: Most of the CHD diagnoses were made when the patients were very young, nearly half of them being diagnosed in infancy, and an extra 10.9% during the neonatal period. However, a considerable part of the population only gets diagnosed during adulthood.
- Diagnosis age varies by lesion: Ventricular septal defect (VSD) was predominant among infants, whereas atrial septal defect (ASD) was frequently identified in adults and D-transposition of the great arteries (D-TGA) and hypoplastic left heart syndrome in newborns.
- Ventricular septal defect and Tetralogy of Fallot (TOF) are most frequent: In general, among the acyanotic lesions, VSD was the most commonly observed congenital heart defect. Of the cyanotic lesions, TOF was the one that occurred the most.
- Gender differences exist for specific CHDs: Even though the total sample had almost identical numbers of males and females, certain illnesses demonstrated marked gender predominance: girls were the majority among cases of ASD and patent ductus arteriosus, whereas aortic stenosis, D-TGA, and coarctation were found mainly in males.

## Introduction

Congenital heart disease (CHD) is the most common type of human birth defect in the world. Congenital heart disease is described as defects of the heart or great vessels that are present at birth.^1,2^ The global prevalence rate is estimated to be approximately 1% of live births,^3^ thus reflecting a significant public health issue affecting the population, specifically the infant and young children, characterized by elevated morbidity and mortality.^4,5^ Although innovations and discoveries in medical science, including advancements in diagnostics and surgical skills, have been extensive,^6^ CHD remains one of the leading contributors to death in infants and young children owing to non-communicable diseases.^4,7^

The global burden of CHD is large, with estimates about 1.35 million children are born with CHD each year.^5,^^7^Notably, the burden of disease is not equally shared, revealing important inequalities by socioeconomic status.^7,8^ Those in low and low-middle socio-demographic index (SDI) regions experience the greatest rates of prevalence and mortality,^7,8^ indicative of continuing inequities in healthcare access and healthcare quality.^9^ For example, while global rates of CHD mortality fell between 1990 and 2021,^7^ improvements in mortality have lagged in regions with low SDI.^10,11^

The severity of CHD lesions is heterogeneous, impacting the clinical presentation and time of diagnosis. Severe types may require complicated surgery and long-term care.^12^ They usually present after birth with significantly severe symptoms such as respiratory distress or cyanosis.^13^ Critical defects (e.g., complete transposition of the great arteries (TGA) or hypoplastic left heart syndrome (HLHS)) can frequently be diagnosed through prenatal imaging and will likely require immediate surgery after birth.^12^ Untreated CHD of a severe type will likely lead to heart failure, failure to thrive, and early death.^14^ In fact, a person with CHD is more likely to die due to their condition before 1 year old, and most of the deaths and disability-adjusted life years are attributable to infants aged less than 12 months, with a substantial proportion before 1 month old.^7,10^

On the other hand, less severe types of CHD, such as atrial septal defects (ASD) and patent ductus arteriosus (PDA), may have a delayed presentation of symptoms in infancy or childhood^15^ and, in some cases, go undiagnosed until adulthood.^16^ This dilemma highlights one of the key challenges of studying CHD in an epidemiologic context: most studies find that the most common lesion worldwide is ventricular septal defect (VSD);^17,18^ however, the detection of lesions that are less severe is contingent upon the availability and utilization of diagnostic technology (i.e., echocardiography).^19^

In addition to classifying severity, understanding demographic patterns, notably age and sex differences, is becoming increasingly important for personalized care and prevention.^20^ The medical literature routinely acknowledges the role of sex in CHD epidemiology, with researchers finding a significantly increased risk for males to be born with more severe CHD, specifically with lesions that involve the outflow tract, such as aortic stenosis (AS), coarctation of the aorta (CoAo), and TGA.^20^ On the other hand, females are more likely to have had milder CHD subtypes that are generally lesions affecting the inflow tract, such as ASD and PDA.^20^

Given this context of persistent global inequalities and differential presentation patterns, detailed regional analyses focusing on age- and sex-specific diagnostic trends are essential. This study, examining 2221 CHD patients, seeks to enhance the essential knowledge base by accurately delineating the epidemiological spectrum of CHD within this cohort in Iraq. The findings reveal a significant predominance of acyanotic lesions and highlight crucial disparities in the age at diagnosis and sex distribution across specific lesion types, thereby emphasizing the urgent necessity for early screening and tailored clinical awareness.

## Material and Methods

### Study Design and Sampling

The study included a total of 2221 patients who attended the outpatient clinic in Ibn Al-Bitar Specialized Center For Cardiac Surgery, Baghdad, Iraq. In this regard, the patients who attended the above-mentioned hospital underwent comprehensive medical and clinical investigations for inclusion in the study. The age range of these patients extended from neonates to adults in a purposive way.

### Setting and time

This study was conducted in Ibn Al-Bitar Specialized Center for Cardiac Surgery inBaghdad, Iraq. The study period lasted for 1 year, extending from September 2022 to September 2023. It received patients who have suspected or established heart diseases from all over the country. All the required investigations for the study were carried out at Ibn Al-Bitar Specialized Center For CardiacSurgery.

Ibn Al-Bitar Specialized Center for CardiacSurgery is considered the major cardiac center in Iraq. It receives more than 250 patient visits daily. The services include cardiac catheterization for both adult and pediatric patients, as well as surgical procedures. The center has a capacity of 150 inpatient beds. It is located in Al-Salhiya, Baghdad, and employs approximately 1400 personnel, including both medical and non-medical staff.

### Data Collection and Measures

The data were recorded at presentation and included the patient’s age, gender, and the final diagnosis. The following age groups were identified for categorization: Neonates: 1 day to less than 28 days (1 to <28 days); Infants: 29 days to less than 2 years (<2 years); Preschool children: from 2 years to less than 6 years (<6 years); school children: from 6 years to less than 12 years (<12 years); adolescents: greater than 12 years (>12 years); and adults: greater than 18 years (>18 years).

### Inclusion and Exclusion Criteria

Cases were included following the clinical diagnosis of CHD. Congenital heart disease was defined according to American Heart Association guidelines^21^ as the structural heart or intrathoracic great vessel disease that is actually or potentially of functional significance present at the time of birth, even if there was a delay in detection. The analysis excluded several specific anomalies, including the anomalies of position and laterality, the right aortic arch, the bicuspid aortic valve, and mitral valve prolapse. Patients with acquired heart diseases, such as cardiomyopathy, rheumatic heart diseases, and functional changes like tricuspid, mitral, pulmonary, and aortic insufficiencies, were also excluded.

### Procedure

To guarantee a conclusive diagnosis of CHD, a series of tests were conducted on each patient. Patient history, clinical evaluation, oxygen saturation, chest X-ray, electrocardiogram, and echocardiograms were all part of these assessments.

Investigational procedure details included:

- Radiographers performed chest radiographs, which a pediatric cardiologist then reported.
- The MAC 500 machine was used to obtain electrocardiograms at a paper speed of 25 mm/second.
- Philips CX50 and Vivid E9 machines were used to obtain echocardiograms, which pediatric cardiologists then interpreted.

### Diagnosis

The American Heart Association definition served as the foundation for the clinical diagnosis of CHD. The sequential analysis of the performed echocardiogram was used to determine the final classification of CHD. The Congenital Heart Surgery Nomenclature and Database Project^22^ and the European Pediatric Cardiac Code^23^ were followed in terms of nomenclature. The Congenital Heart Surgery Nomenclature and Database Project (Mavroudis C) and the European Pediatric Cardiac Code (Franklin RCG) nomenclature were used to classify CHD based on the sequential analysis of the performed echocardiogram.

### Statistical Analyses

The normality of the numerical variables in the patients was confirmed using a Q-Q norm plot. The mean (standard deviation) and number (percentage) were used for the descriptive statistics. The mean age differences of the types of CHD were examined in an independent *t*-test. The prevalence of CHD in different age groups was presented in number (percentage). The different types of CHD according to age at diagnosis were presented in number (percentage). The gender difference in all types of CHDs was examined in the chi-squared test. The prevalence of acyanotic and cyanotic CHD lesions was presented in number (percentage). The significant level of difference was determined at *P* < .05. The statistical calculations were performed using IBM SPSS Statistics for Windows (Version 20.0), Armonk, NY: IBM Corp.

### Ethical Approval

The ethical approval of this study was obtained from the local health research ethics committee in Baghdad and was registered as number 16452/6 in June 2022. This study protected the confidentiality of the patients’ personal information. The informed consent from all patients was obtained before including them in the study.

## Results

Table 1 presents the overall distribution of CHD in relation to patient age. Out of a total of 2221 patients, 1704 (76.7%) had acyanotic CHD, while 517 (23.3%) had cyanotic CHD. The mean age of patients with acyanotic CHD was much higher (81.3 months) than those with cyanotic CHD (25.6 months; Table 1).

Table 2 focuses specifically on the age of the diagnosis of CHD. Nearly half of all cases (47.5%) were diagnosed during infancy, and another 10.9% were identified in the neonatal period, illustrating the value of early detection in the first 2 years of life. Preschool children (2-6 years) accounted for 14.7% of diagnoses, while school-aged children (6-12 years) were 9.2%. A substantial group of patients (13.2%) were only diagnosed in adulthood (Table 2).

Table 3 provides a detailed breakdown of each specific type of CHD according to the age at which it was diagnosed. The most common lesion, VSD, was diagnosed across all age groups, with the highest proportion in infants (49.7%). Atrial septal defect had a completely unique pattern, with almost 42% of cases identified only in adulthood, reflecting the silent nature of ASD and its tendency to go unnoticed until complications appear. Critical lesions such as D-transposition of the great arteries (D-TGA) and HLHS were mostly diagnosed in neonates (61.4% and 55.6%, respectively). Conditions like Tetralogy of Fallot (TOF) and PDA were mainly diagnosed in infancy, but some were detected later in school age or adolescence (Table 3).

The table 4 presents the gender distribution of different types of CHDs among 2221 patients. Overall, the sample was almost equally divided between males (50.5%) and females (49.5%), giving a male-to-female ratio of 1.02 : 1. However, specific CHD types showed distinct gender patterns. Interventricular septal defect, the most common condition (670 cases), had a nearly equal distribution, with a slight male predominance that was not statistically significant. In contrast, interatrial septal defect was significantly more common in females (66.5% vs. 33.5% in males, *P* < .0001), with a male-to-female ratio of 1 : 1.9. Similarly, patent ductus arteriosus was also more frequent in females (62.1% vs. 37.9%, *P* = .0002). On the other hand, several conditions showed significant male predominance: aortic stenosis (80.7% males, *P* < .0001, ratio 4.1 : 1), D-transposition of the great arteries (66.3% males, *P* = .0035), coarctation (61.5% males, *P* = .0191), and total anomalous pulmonary venous drainage (78.6% males, *P* = .0352). Other conditions, such as TOF, atrioventricular septal defect, tricuspid atresia, and single ventricle anomalies, did not demonstrate significant gender differences, although some had slight trends. Rare anomalies such as Ebstein anomaly, congenitally corrected transposition, and hypoplastic left heart syndrome were too infrequent to show consistent gender differences (Table 4).

Table 5 summarizes the prevalence of different acyanotic and cyanotic lesions. Among acyanotic CHDs, VSD was the most frequent (30.2% of all cases), with subtypes such as perimembranous VSD, inlet VSD, outlet VSD, and muscular VSD listed. Atrial septal defect was the second most common (12.8%), followed by pulmonary stenosis (10%) and PDA (9.1%). Together, these acyanotic lesions constituted more than three-quarters (76.7%) of all cases. Cyanotic CHDs were less common (23.3%), but TOF was the most common (9.4%), followed by transposition of the great arteries (3.7%) and double outlet right ventricle (2.1%). Less frequent but clinically important lesions such as tricuspid atresia, single ventricle, Ebstein anomaly, and hypoplastic left heart syndrome were also present, though in small numbers (Table 5).

## Discussion

The analysis of 2221 CHD patients yields crucial, multifaceted insights regarding disease prevalence, diagnostic timing, and demographic distribution, findings that resonate strongly with established international epidemiological and clinical patterns. The study confirms a typical distribution of physiological groups and specific lesions while accentuating the differential challenges posed by severe versus mild defects in achieving timely diagnosis.

### Spectrum and Physiological Classification

The study showed that acyanotic CHD was much more common than cyanotic CHD, with 76.7% of cases being acyanotic and only 23.3% being cyanotic. This high proportion of acyanotic defects is internationally consistent, reflecting the fact that simple shunt lesions generally constitute the largest fraction of detected congenital cardiac anomalies.^24,25^ For instance, a study among Egyptian children reported acyanotic CHD in 79.2% of their cohort.^25^ Ventricular septal defect accounted for 30.2% of all cases in the study, making it the most prevalent lesion within the acyanotic category. The study’s results are consistent with the worldwide view that VSD is the most common type of CHD.^9,16^ Among acyanotic defects, the prevalence rates were as follows: PDA was 9.1%, pulmonary stenosis (PS) was 10%, and ASD was 12.8%. The prevalence of septal defects was further supported by the fact that in a different Baghdad cohort, VSD accounted for 28.3% of diagnoses, followed by ASD (19.7%) and PDA (15.4%).^18^

In the cyanotic group, TOF was the most frequent lesion, accounting for 9.4% of all CHD cases in the cohort, followed by transposition of the great arteries (3.7%) and double outlet right ventricle (2.1%). Tetralogy of Fallot’s leading position among cyanotic defects is widely reported across various geographies, including Central India^24^ and Egypt.^25^ The distribution observed here, with complex cyanotic lesions constituting a significant minority (23.3%), aligns generally with reports where complex CHD comprised around 18.5% of cases.^18^

### The Age at Diagnosis

One of the most striking findings is the significant disparity in the mean age at diagnosis between the 2 physiological groups. Patients with cyanotic CHD were diagnosed at a mean age of 25.6 months, dramatically earlier than patients with acyanotic CHD, whose mean age at diagnosis was 81.3 months. This divergence underscores the fundamental difference in clinical presentation driven by lesion severity.^13^ Critical defects cause immediate hemodynamic instability, demanding prompt detection.^12^ The study confirmed this principle, primarily identifying critical cyanotic lesions, specifically D-TGA and HLHS, in neonates. The urgency of detecting these lesions is highlighted by the natural history of TGA, where over 90% of affected infants historically died within the first year if untreated.^26^ Overall, the cohort demonstrated moderate success in early detection, with nearly 58% of all cases diagnosed by age 2 (10.9% neonatally, 47.5% in infancy). Ventricular septal defect diagnosis was concentrated in infancy (49.7%). However, the persistence of delayed diagnosis, particularly for milder defects, remains a substantial challenge.

### The Problem of Delayed Diagnosis and Adulthood Presentation

A significant portion of the cohort, 13.2%, was diagnosed only in adulthood. This delay was strongly associated with specific shunt lesions: ASD frequently went undetected until later life, with 42% of cases diagnosed in adulthood. Milder defects like ASD and PDA are known globally for their potential to be diagnosed late, sometimes only presenting symptoms in childhood or adulthood.^15,16^ In resource-constrained environments, delayed diagnosis is amplified, contributing to poor outcomes.^27^

For instance, due to a lack of specialized centers, regional studies in Iraq have found that CHD is frequently diagnosed late, often in adulthood.^28^ Worldwide, the prevalence of mild defects like ASD and VSD has increased due to the use of specialized tools like echocardiography.^19^ The prevalence of first-time diagnoses of CHD has decreased in recent decades, 19 indicating a positive shift toward earlier detection, even though the rise in prevalence of CHD in children is associated with the increased recognition of milder cases.^29^ However, the observation of late diagnoses for ASD supports the continued need for consistent screening initiatives that continue into childhood and adolescence.

### Distinct Gender-Specific Patterns

With a male-to-female ratio of 1.02 : 1, the overall gender distribution was almost equal. This ratio is consistent with other population reports in the region, including the 1.18 : 1 ratio in a Baghdad study^18^ and the 1.2 : 1 ratio observed among Egyptian children.^25^ However, examining specific lesions reveals profound sex-based biological differences consistent with extensive literature on CHD pathogenesis.^20^

The study found a strong male predominance in lesions typically associated with severity or outflow tract anomalies: AS, D-TGA, coarctation, and total anomalous pulmonary venous drainage (TAPVD).

This pattern corroborates the established observation that males are at an elevated risk for severe CHD^20^ and defects associated with left ventricular outflow tract obstruction, including AS, CoAo, and HLHS. Transposition of the great arteries, specifically, shows a consistent male predilection in multiple cohort studies.

On the other hand, females were more likely to have defects like ASD and PDA, which are usually shunt lesions or inflow tract anomalies. The notion that inflow tract defects are more common in females is supported by the extensive documentation of this pattern in the literature on CHD.^30,31^ Research has consistently shown that women are more likely than men to have ASDs, and this distribution pattern is frequently seen regardless of the patient’s age.^31^

These consistent sex-specific distributions demonstrate that CHD is a spectrum disease with a complex etiology that likely involves a complex interaction of environmental, hormonal, and genetic factors that affect cardiac development.^32^

### Policy Implications and the Need for Targeted Interventions

Improved public health strategies are desperately needed, as evidenced by the high mortality burden concentrated in neonates,^10^ the prevalence of defects that allow for delayed diagnosis, and the persistent health disparities in low SDI settings.^9^

The study emphasizes the need for customized clinical awareness and early screening. Better delivery planning and prompt postnatal intervention are made possible by enhanced prenatal detection of critical lesions through fetal echocardiography.^33^ Comprehensive follow-up systems are required to ensure that mild or asymptomatic cases do not result in late complications, especially for milder defects like ASD that account for significant adult diagnoses.^29^

Deficits in specialized medical facilities and inadequate prenatal care continue to exist in resource-constrained regions, such as those addressed by parallel studies pertaining to the Middle East and North Africa region.^34^ To reduce late presentation and high mortality rates, especially in the neonatal period, it is imperative to invest in pediatric cardiology infrastructure, increase diagnostic capacity (such as Co/ov2 Echocardiography), and enhance early detection programs (such as pulse oximetry screening). Furthermore, improving outcomes for kids with CHD requires tackling systemic obstacles like a lack of qualified professionals and weak referral systems in developing nations.^28^ Healthcare systems around the world can shift toward a more equitable and efficient management approach by prioritizing resource allocation based on the known age- and sex-specific patterns of CHD.^20^

This thorough analysis of 2221 CHD patients reveals different epidemiological patterns that are essential for establishing future healthcare priorities. Ventricular septal defect was the most common lesion overall, and the cohort showed a significant majority of acyanotic CHD over cyanotic CHD. The severity of the defect had a significant impact on when the condition was diagnosed; the mean age of diagnosis for cyanotic CHD was 25.6 months, while the mean age of diagnosis for acyanotic CHD was 81.3 months. Since most CHD-related deaths occur in the crucial early days of life, critical cyanotic lesions like D-TGA and HLHS were primarily found in neonates. On the other hand, less severe lesions—more specifically, ASD—often remained undiscovered until adulthood, confirming the well-known difficulties in detecting asymptomatic shunt defects. Significant sex-specific patterns were also verified: complex outflow tract anomalies (e.g., aortic stenosis, D-TGA) showed a strong male predominance, while lesions like ASD and PDA showed a female predominance. These detailed age- and sex-specific findings reinforce the urgent need for enhanced early screening and tailored clinical awareness to address delayed diagnoses and improve outcomes.

## Figures and Tables

**Table 1.** Types of CHD in Relation to Patients’ Age

**Congenital Heart Disease (n = 2221)**	**Age (Months)** **Mean (SD)**	**No.**	**%**	***P***
Acyanotic CHD	81.34 (107.34)	1704	76.7	<.001
Cyanotic CHD	25.59 (50.14)	517	23.3	
Total No.	68.51 (100.28)	2221	100	

CHD, congenital heart disease; SD, standard deviation.

**Table 2.** Age at Diagnosis of CHD in Iraqi Patients

**Age Groups**	**Age (Months)** **Mean (SD)**	**No.**	**%**
Neonates* (1-<28 days)	11.38 (3.75)	243	10.9
Infant (29 days-<2 years)	11.72 (6.50)	1055	47.5
Preschool (2-<6 years)	44.79 (11.37)	327	14.7
School Child (6-<12 years)	105.66 (24.29)	205	9.2
Adolescent (12-<18 years)	171.71 (16.58)	98	4.4
Adult (≥18 years)	294.60 (61.37)	293	13.2
Total	68.51 (100.28)	2221	100

*Neonatal mean age accounted in days.

SD, standard deviation.

**Table 3.** Different Types of CHD According to Age at Diagnosis

**CHD**	**Statistics No. (%)**						
	**Neonates **	**Infant**	**Preschool Child **	**School Children**	**Adolescent **	**Adult**	**Total**
VSD	65 (9.7)	333 (49.7)	112 (16.7)	77 (11.5)	31 (4.6)	52 (7.8)	670 (100)
ASD	14 (4.9)	65 (22.9)	35 (12.3)	26 (9.2)	25 (8.8)	119 (41.9)	284 (100)
PS	7 (3.1)	109 (48.9)	32 (14.3)	16 (7.2)	16 (7.2)	43 (19.3)	223 (100)
TOF	14 (6.7)	112 (53.6)	34 (16.3)	30 (14.4)	6 (2.9)	13 (6.2)	209 (100)
PDA	4 (2.0)	113 (55.7)	44 (21.7)	23 (11.3)	4 (2.0)	15 (7.4)	203 (100)
COA	8 (7.3)	37 (33.9)	19 (17.4)	11 (10.1)	8 (7.3)	26 (23.9)	109 (100)
AS	2 (2.3)	29 (33.0)	16 (18.2)	11 (12.5)	8 (9.1)	22 (25.0)	88 (100)
D-TGA	51 (61.4)	30 (36.1)	1 (1.2)	1 (1.2)	0	0	83 (100)
AV septal defect	14 (18.2)	49 (63.6)	10 (13.0)	4 (5.2)	0	0	77 (100)
DORV	11 (23.9)	33 (71.7)	2 (4.3)	0	0	0	46 (100
SV	14 (28.0)	35 (70.0)	1 (2.0)	0	0	0	50 (100)
TA	8 (32.0)	16 (64.0)	1 (4.0)	0	0	0	25 (100)
PA with VSD	10 (43.5)	12 (52.2)	1 (4.3)	0	0	0	23 (100)
EA	0	13 (81.3)	1 (6.3)	2 (12.5)	0	0	16 (100)
CC TGA	1 (6.3)	8 (50.0)	5 (31.3)	2 (12.5)	0	0	16 (100)
PAIS	2 (12.5)	14 (87.5)	0	0	0	0	16 (100)
PTA	8 (53.3)	7 (46.7)	0	0	0	0	15 (100)
TAPVD	1 (7.1)	13 (92.9)	0	0	0	0	14 (100)
MA	3 (25.0)	9 (75.0)	0	0	0	0	12 (100)
CMVD	0	2 (20.0)	5 (50.0)	2 (20.0)	0	1 (10.0)	10 (100)
CAA	0	4 (44.4)	3 (33.3)	0	0	2 (22.2)	9 (100)
HLHS	5 (55.6)	4 (44.4)	0	0	0	0	9 (100)
PAPVD	0	2 (40.0)	3 (60.0)	0	0	0	5 (100)
AP window	0	5 (100)	0	0	0	0	5 (100)
Cortriatriatum	1 (25.0)	1 (25.0)	2 (50.0)	0	0	0	4 (100)
Total No.	243	1055	327	205	98	293	2221

AAO, ascending aorta; AO, aorta; APW, aortopulmonary window; AS, aortic stenosis; ASD, atrial septal defect; AV, Atrioventricular; CAA, coronary artery anomaly; CCTGA, congenitally corrected transposition of great arteries; CHD, congenital heart disease; CMVD, congenital mitral valve disease; COA, coarctation of aorta; DAO, descending aorta; DORV, double outlet right ventricle; D-TGA, transposition of great arteries; EA, Ebstein’s anomaly; HLHS, hypoplastic left heart syndrome; IVC, inferior vena cava; LA, left atrium; LV, left ventricle; MA, mitral atresia; MPA, main pulmonary artery; MR, mitral regurgitation; PA with VSD, pulmonary atresia with ventricular septal defect; PA, pulmonary artery; PAIS, pulmonary atresia with intact septum; PAPVD, partial anomalous pulmonary venous drainage; PCWP, pulmonary capillary wedge pressure; PDA, patent ductus arteriosus; PFC, persistent fetal circulation; PFO, patent foramen ovale; PHT, pulmonary hypertension; PS, pulmonary stenosis; PTA, persistent truncus arteriosus; PVOD, pulmonary vascular obstructive disease; PVR, pulmonary vascular resistance; RA, right atrium; RV, right ventricle; SV, single ventricle; SVC, superior vena cava; SVR, systemic vascular resistance; TA, tricuspid atresia; TAPVD, total anomalous pulmonary venous drainage; TOF, tetralogy of Fallot; VSD, ventricular septal defect.

**Table 4.** Gender Difference in All Types of CHDs

**CHD (n = 2221)**	**All Patients ** **No. (%)**	**Gender No. (%)**		***P***	**M/F Ratio**
		**Male**	**Female**		
Interventricular septal defect	670	354 (52.8)	316 (47.2)	.151	1.1 : 1
Interatrial septal defect	284	95 (33.5)	189 (66.5)	<.001	1 : 1.9
Pulmonary stenosis	223	101 (45.3)	121 (54.7)	.115	1 : 1.1
Tetralogy of Fallot	209	119 (56.9)	91 (43.1)	.061	1.3 : 1
Patent ductus arteriosus	203	77 (37.9)	126 (62.1)	.000	1 : 1.6
Coarctation	109	67 (61.5)	42 (38.5)	.019	1.5 : 1
Aortic stenosis	88	71 (80.7)	17 (19.3)	<.001	4.1 : 1
D-TGA	83	55 (66.3)	28 (33.7)	.004	1.9 : 1
Atrioventricular septal defect	77	32 (41.6)	45 (58.4)	.110	1 : 1.4
DORV	46	27 (58.7)	19 (41.3)	.262	1.4 : 1
Single ventricle	50	30 (60.0)	20 (40.0)	.175	1.5 : 1
Tricuspid atresia	25	8 (32.0)	17 (68.0)	.102	1 : 2.1
Pulmonary atresia with VSD	23	13 (56.5)	10 (43.5)	.563	1.3 : 1
Ebstein anomaly	16	7 (43.8)	9 (56.3)	.587	1 : 1.2
Congenitally corrected transposition	16	11 (68.8)	5 (31.3)	.1433	2.2 : 1
Pulmonary atresia with intact septum	16	6 (37.5)	10 (62.5)	.296	1 : 1.6
Persistent truncus arteriosis	15	9 (60.0)	6 (40.0)	.461	1.5 : 1
TAPVD	14	11 (78.6)	3 (21.4)	.035	3.6 : 1
Mitral atresia	12	8 (66.7)	4 (33.3)	.262	2 : 1
Congenital mitral valve disease	10	5 (50.0)	5 (50.0)	.974	1 : 1
Coronary artery anomaly	9	4 (44.4)	5 (55.6)	.715	1 : 1.2
Hypoplastic left heart syndrome	9	5 (55.6)	4 (44.4)	.762	1.2 : 1
Partial anomalous PVD	5	2 (40.0)	3 (60.0)	.638	1 : 1.5
AP window	5	3 (60.0)	2 (40.0)	.671	1.5 : 1
Cortriatriatum	4	2 (50.0)	2 (50.0)	.984	1 : 1
Total No.	2221	1122 (50.5)	1099 (49.5)		1.02 : 1

AP, Aortopulmonary; CHD, congenital heart disease; DORV, double outlet right ventricle; D-TGA, D-transposition of the great arteries; PVD, peripheral vascular disease; TAPVD, total anomalous pulmonary venous drainage; T-DGA, transposition of great arteries; VSD, ventricular septal defect.

**Table 5.** Prevalence of Acyanotic and Cyanotic CHD Lesions

**CHDs**	**Type of the Defect**	**No. (%)**	**Total No.**	**%**
A cyanotic CHD				
Ventricular septal defect	PM VSD Inlet VSD Outlet VSD Muscular VSD	554 (82.6) 30 (4.5) 23 (3.4) 64 (9.5)	670	30.2
Atrial septal defect (Isolated)	ASD secondum Sinus Venosus	258 (90.8) 26 (9.2)	284	12.8
Pulmonary stenosis			223	10.0
Patent ductus arteriosus (Isolated)			203	9.1
Coarctation (Isolated)			109	4.9
Aortic stenosis			88	4.0
Atrioventricular septal defect	Complete AV canal Partial AV canal	28 (36.4) 49 (63.6)	77	3.5
Congenital mitral valve disease			10	0.5
Coronary artery anomaly			9	0.4
AP window			5	0.2
Partial anomalous PVD			5	0.2
Cortriatriatum			4	0.2
Subtotal No.			1704	76.7
Cyanotic CHD				
Tetralogy of Fallot			209	9.4
D-TGA	Simple D-TGA	49 (59.0)	83	3.7
	Complex D-TGA	34 (41.0.)		
DORV	DORV with PS DORV without PS	32 (69.6) 14 (30.4)	46	2.1
Single ventricle			50	2.3
Tricuspid atresia			25	1.1
Pulmonary atresia with VSD			23	1.0
Ebstein anomaly			16	0.7
Congenitally corrected TGA			16	0.7
PAIS			16	0.7
Persistent truncus arteriosis			15	0.7
TAPVD			14	0.6
Mitral atresia			12	0.5
Hypoplastic left heart syndrome			9	0.4
Subtotal No.			517	23.3
Total No.			2221	100

ASD, atrial septal defect; CHD, congenital heart disease; DORV, double outlet right ventricle; D-TGA, D-transposition of the great arteries; PAIS, pulmonary atresia with intact septum; PS, pulmonary stenosis; TAPVD, total anomalous pulmonary venous drainage; , VSD, ventricular septal defect.

## Data Availability

The data that support the findings of this study are available on request from the corresponding author.
